# Immunostimulating Effect of Inactivated Parapoxvirus Ovis on the Serological Response to Equine Influenza Booster Vaccination

**DOI:** 10.3390/vaccines10122139

**Published:** 2022-12-14

**Authors:** Flora Carnet, Romain Paillot, Christine Fortier, Erika S. Hue, Laurie Briot, Frédéric de Geoffroy, Pierre-Olivier Vidalain, Stéphane Pronost

**Affiliations:** 1 LABÉO, 14280 Saint-Contest, France; 2 BIOTARGEN, Normandie University, UNICAEN, 14280 Saint-Contest, France; 3 School of Equine and Veterinary Physiotherapy, Writtle University College, Lordship Road, Writtle, Chelmsford CM1 3RR, UK; 4 Institut Français du Cheval et de l’Equitation, Plateau Technique du Pin-au-Haras, 61310 Gouffern en Auge, France; 5 CIRI Centre International de Recherche en Infectiologie, Univ. Lyon, Inserm, U1111, Université Claude Bernard Lyon 1, CNRS, UMR5308, ENS de Lyon, 69007 Lyon, France

**Keywords:** horse, equine influenza viruses, vaccine, immunomodulator, inactivated Parapoxvirus ovis

## Abstract

Equine influenza virus (EIV) is responsible for recurring outbreaks that are detrimental to the equine industry. Vaccination is key for prevention, but the effectiveness and duration of protection provided by existing vaccines is often insufficient. In order to improve vaccine efficacy, we evaluated the benefit of immune stimulation with inactivated Parapoxvirus ovis (iPPVO) on the antibody response induced by a vaccine boost against EIV. A whole inactivated ISCOMatrix-adjuvanted equine influenza vaccine was administered alone (*n* = 10) or combined with iPPVO injections at D0, D2 and D4 post vaccination (*n* = 10) to adult horses that required a vaccine boost 6 months after the last immunization, as now recommended by the WOAH. Antibody levels were measured with the single radial haemolysis (SRH) assay at 1, 3 and 6 months post-vaccination. Results revealed that horses that received iPPVO had higher antibody levels than the control group injected with the EI vaccine alone. Although the vaccine used contains only a clade 1 and European lineage strain, the increase in protective antibodies was also observed against a clade 2 strain. Thus, immune stimulation with iPPVO, a substance already marketed as an immunostimulant, could be used to improve vaccination protocols in horses and potentially other species.

## 1. Introduction

Equine influenza virus (EIV) is an Alphainfluenzavirus of the Orthomyxoviridae family. It is the etiological agent of horse flu or equine influenza (EI), a highly contagious disease of the respiratory tract that is transmitted from equine to equine mainly by contaminated aerosols. The main clinical signs are fever, nasal discharge, coughing, loss of appetite and general weakness. Complications include pulmonary oedema, asthma and secondary bacterial infection. In rare cases, EI eventually leads to the death of the animal [1,2]. EIV still causes major outbreaks worldwide despite surveillance and prevention measures [3]. In 2007, Australia faced an outbreak of EI in a naïve horse population that cost over AUS 1 billion to the equine industry and the government [4,5,6]. In 2019, Europe was confronted by a major circulation of EIV, with multiple outbreaks in France, Belgium and Germany. The UK alone reported over 200 outbreaks of EI, causing the shutdown of horse racing for 6 days with considerable economic losses [4]. Over the 2010–2021 period, the number of outbreaks substantially increased in North and South America, Europe, Asia and Africa [7].

The most widely used prophylactic method to limit the spread of the disease is vaccination. Vaccination has two major aims: to reduce the duration and intensity of clinical signs of disease and to limit the transmission of the virus within a population of horses. Many vaccines are commercially available worldwide and are capable of inducing protective immunity [2]. The surface hemagglutinin (H) and neuraminidase (N) are among the main immunodominant antigens of influenza viruses. EIV strains responsible for current outbreaks belong to the H3N8 subtype (subtype A2), whereas the H7N7 subtype of EIV (subtype A1) has not been isolated since 1979 [3,8]. Like all influenza viruses, EIV mutates over time and thus escapes host immunity. Since the 1980s, this virus has evolved into two lineages: the European lineage and the American lineage. The American lineage has itself evolved into sub-lineages and clades, resulting in a clade 1 and a clade 2 in the Florida sub-lineage (FC1 and FC2). Clade 2 viruses of the Florida sub-lineage were responsible for most outbreaks in Europe from 2005 to 2018 [3]. The evolution of EIV has led the WOAH to recommend vaccine updates to remain effective. Despite this, a large majority of vaccines do not contain the recommended strains to protect against circulating EIV strains [7]. In addition to this genetic drift that tends to decrease the efficacy of vaccines, protective antibody levels are rarely achieved after a single shot of vaccine, and three injections are usually necessary. Furthermore, because antibody levels tend to wane with time, booster injections are recommended every 6 months or yearly depending of the activity and/or risk of infection. Vaccination is, therefore, important to prevent the spread of the virus [2], but it is critical to keep vaccines up-to-date and to maintain high levels of antibodies in equine populations. 

One option to improve the level and duration of protection is to stimulate the innate immune response during vaccination, as it will determine the quality of the adaptive immune response that is induced. Inactivated Parapoxvirus ovis or iPPVO is an immunostimulant marketed as Zylexis by Zoetis^®^ and is composed of the inactivated Parapoxvirus ovis strain D1701 (iPPVO). This compound is recommended for prophylactic and metaphylactic treatment of infectious diseases in horses, cattle, pigs, dogs and cats [9,10,11,12,13]. Previous studies suggested that iPPVO reduces secondary viral or bacterial shedding in horses; however, the numbers of horses involved were small [9,14]. Injection of iPPVO results in a non-specific stimulation of the innate immune response [15]. The activity of iPPVO has been mainly associated with the synthesis of the early pro-inflammatory cytokines IL-6, IL-8, TNF alpha, IL-2, and IFN and the Th1 cytokines IL-12, IL-18 and IFN gamma [16,17]. Hue et al., showed the efficacy of iPPVO in vitro as assessed by rapid and strong IFN type I and II responses in the context of equine PBMC infection with EHV1 and EHV4 [18]. iPPVO has been shown to have immunomodulatory activity (increased innate immune cells and neutralising antibodies) in several diseases: genital herpes in guinea pigs, human hepatitis B virus infection in transgenic mice, pseudorabies virus infection in mice, Aujeszky’s disease in pigs and infectious bovine rhinotracheitis [10,16,19,20]. Because iPPVO is an approved immunostimulant in veterinary medicines, we studied the potential beneficial effect of iPPVO injections on the immunological response of horses to a booster vaccination against EI. It should be noted that in our experimental design, iPPVO was not mixed and co-administered with the vaccine, but separately injected within a 15 cm radius of the vaccine injection site.

## 2. Materials and Methods

### 2.1. Ethical Approval/Animal Welfare

This study was authorised by the CENOMEXA n°54 ethics committee (N° APAFIS#29210-2020113009246927 v3) and the Ministry of Higher Education, Research and Innovation.

### 2.2. Horses and Screening Process

The study was carried out at the Jumenterie du Pin in Orne, France between November 2020 and November 2021. A group of 62 horses were screened by single radial haemolysis assay (SRH). Equine influenza SRH antibody titres were measured immediately before as well as 1 and 3 months after the annual EI booster vaccination (Equilis prequenza^®^ Te^®^). The sample size was determined using three methods: the “resource equation method” [21], the Within-factor repeated measures [22] and a method using the Cohen coefficient [23], taking into account the different criteria of this study: two groups and a desired difference of at least 25% between the two groups. Based on these three methods, the optimal number was set to 10 individuals per group. Of the 62 animals, a group of 20 horses with a comparable serological status was assembled according to the following criteria: an SRH titre less than or equal to 200 mm^2^ 3 months after the annual EI booster vaccination and an increase between 10 to 70 mm^2^ in SRH titre between pre-vaccination and 1 month post-vaccination levels. Twenty healthy mares, breed selle-français, anglo-arabian, trotter and draft horses between 11 and 25 years of age were enrolled in the study. Finally, the horses were stratified into three different age groups (i.e., 11–14, 15–18 and 19–25 years) and then randomly allocated to the “control group” that would receive the semi-annual EI booster (SAB) vaccination or the “iPPVO group” that would receive the SAB vaccination plus iPPVO injections.

### 2.3. Study Design

**Figure 1:**
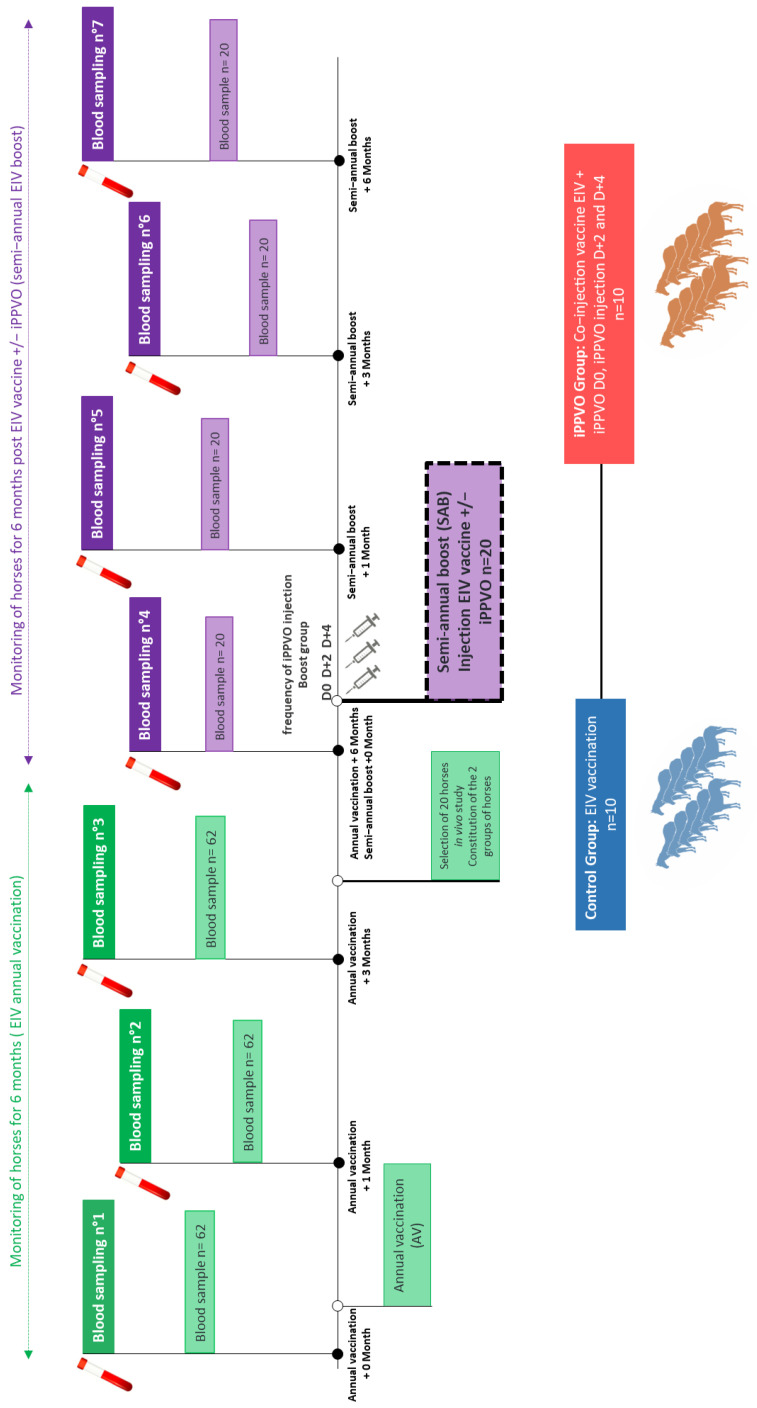
Horse selection and EIV vaccination protocol with or without iPPVO^®^.

Mares in the control group received only the EI vaccine (Equilis prequenza^®^ Te^®^, MSD Animal Health) according to the manufacturer’s recommendations (i.e., 1 mL of suspension injected intramuscularly into the neck). The commercially available Equilis prequenza^®^ Te^®^ inactivated vaccine (MSD Animal Health) contains a virus of the older Eurasian sub-lineage (A/Equi-2/Newmarket/2/93), Florida clade 1 sub-lineage virus (A/equine-2/South Africa/4/03), and the tetanus toxoid adjuvanted with ISCOMatrix. Mares in the vaccine with iPPVO group received one dose of the same EI vaccine and three doses of iPPVO (Zylexis^®^, Zoetis). A batch of Zylexis^®^ produced under commercial conditions was used. The Zylexis^®^ consisted of the lyophilised iPPVO strain D1701. The lyophilised pellet was resuspended in 1 mL of water for injection prior to administration by intramuscular injection (1 mL of solution injected intramuscularly into the neck according to the manufacturer’s recommendations, followed by two new injections of iPPVO at 48 h intervals (i.e., D0, D + 2 and D + 4) (Figure 1). The label information provided with Zylexis^®^ mentioned the use of different volumes depending of the targeted species. As this product had no marketing authorization in France at the time of this study, and this study represents the first use of Zylexis^®^ in combination with another active product (i.e., vaccine), it was decided to use the minimal recommended volume. The injection of iPPVO was performed within a 15 cm radius of the vaccine injection site. After injection, the horses were carefully monitored for side effects for 1 week. Temperature, general condition and local reaction at the injection site were monitored 48 h after the injections. Fever was reported for rectal temperatures > 38.8 °C. No significant side effects from iPPVO injection were observed in relation to the frequency of swelling (Table S1).

### 2.4. Blood Samples

All blood samples (screening and study phases) were taken from the jugular vein (1 dry tube of 10 mL per horse). During the study phase and for both groups, one blood sample was taken immediately prior to boost EI immunisation. Other blood samples were collected 1, 3 and 6 months after the semi-annual boost (SAB) vaccination. All of the samples were stored for a maximum of 4 h at +4 °C before the serum was collected. The collected sera were stored at −20 °C.

### 2.5. Serological Analysis: Single Radial Haemolysis

The SRH assay measures EIV-specific serum antibody levels in horses and was performed according to the WOAH recommendations, 2019 [3]. Briefly, two EIV strains (A/equine/Jouars/4/06 (H3N8; Florida Clade 2) and A/equine/Paris2018 (H3N8; Florida Clade 1)) were used as SRH antigens. Sheep red blood cells were coated with EIV antigens, then incubated at +4 °C for 10 min. Next, a solution of CrCl_3_ (1:400 dilution from dilution stock solution (2.25 M)) was added to the mixture and incubated for 10 min at room temperature. After centrifugation (20 min, 3000 *g*, +4 °C), the supernatant was removed, and the pellet was resuspended in buffered saline. Then, the solution was mixed with an agarose solution and guinea pig complement. The agarose gel was then stamped to make wells into which 10 µL of test and control sera were placed. Sera were previously decomplemented at 56 °C for 30 min. The plates were incubated for 24 h at 42 °C before reading. A clear haemolytic zone appeared around the well when anti-influenza antibodies coupled to complement lyse the antigen-coated red blood cells. The size of the zone was directly proportional to the level of strain-specific antibodies in the serum. A haemolysis area of less than 85 mm^2^ indicates a non-immune horse; between 85 mm^2^ and 150 mm^2^, the horse is clinically protected; above 150 mm^2^, the horse is virologically protected [24,25]. Control antiserum from the European Directorate for the Quality of Medicines and Healthcare (EDQM) was included on each plate (A/equine/South Africa/4/03 Horse antiserum BRP reference Y0000712 for antigen A/equine/Jouars/4/06; a pool of sera positive for strain A/equine/Paris2018). The reading of the SRH results was checked by two readers in order to minimise reading errors. Thirty randomly selected samples were retested by another experimenter. The retested samples showed a difference of less than 7% in the means.

### 2.6. Statistical Data Analysis

Statistical analysis was performed using GraphPad PRISM^®^ 7.04 (San Diego, CA, USA). Normally distributed group samples were analyzed using two-way repeated measures analysis of variance (ANOVA) with Tukey’s multiple comparisons post-test to test significant differences between groups. The frequency of horses reaching the protection threshold in each group was compared at equivalent time points post immunisation (i.e., post-AV time points compared with post-SAB time points) using an χ^2^ test. The frequency of horses with local swelling after injection was also compared using an χ^2^ test. The significance level was set at a *p*-value of <0.05.

## 3. Results

### 3.1. Antibody Response to Annual EIV Vaccination (Pre-iPPVO Treatment)

**Figure 2:**
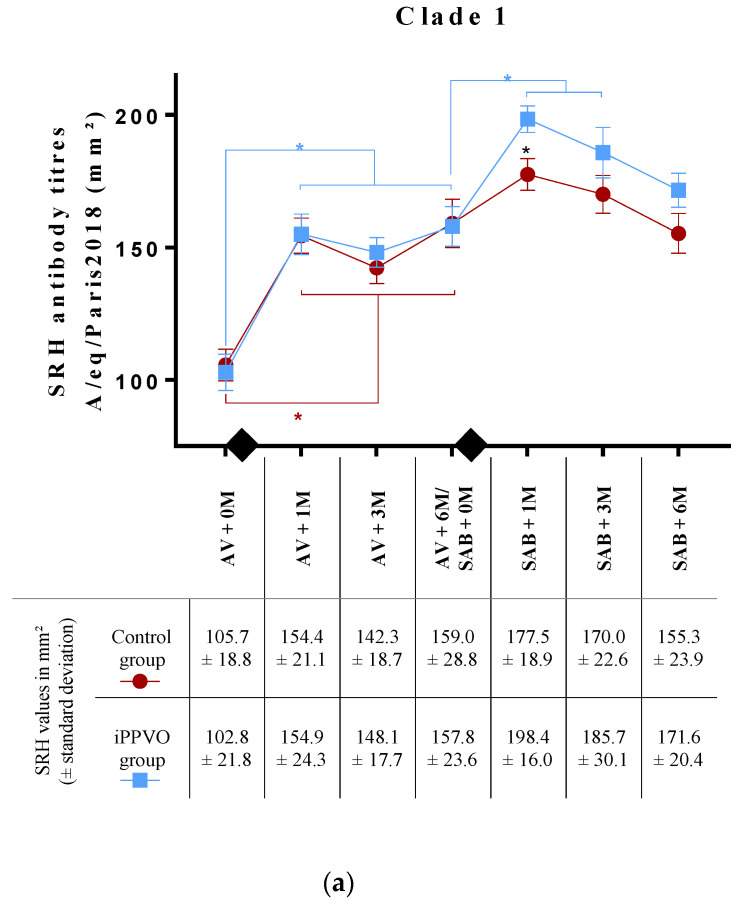

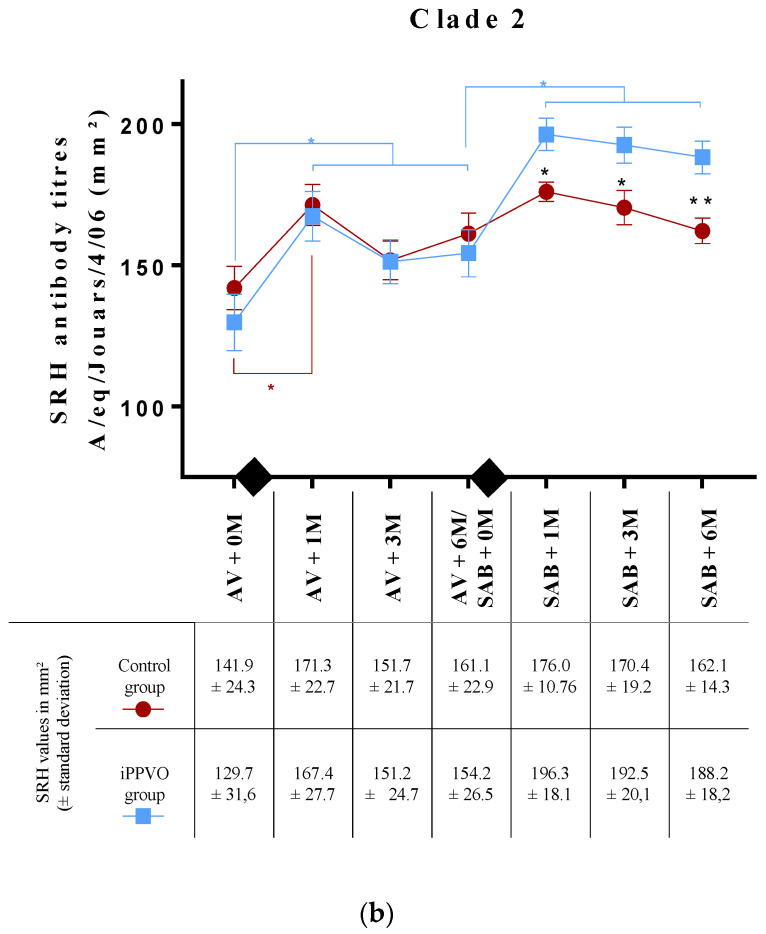
Mean H3N8 single radial haemolysis (SRH) antibody response measured before and after booster vaccination. The values correspond to sera isolated from blood samples that were collected in the control and iPPVO groups before the annual vaccination (AV), 1 and 3 months after the AV and before the semi-annual boost (SAB), and 1, 3 months and 6 months after the SAB. The red circles correspond to the mean SRH values in the control group (*n* = 10), and the blue squares correspond to the mean SRH values in the iPPVO group (*n* = 10) for (**a**) A/equine/Paris/1/2018 strain clade 1 strain and (**b**) A/equine/Jouars/4/06 EIV clade 2 strain. The diamonds represent the time of the AV injection and the 6 months SAB injections with or without iPPVO. Error bars represent standard error of the mean. Black stars correspond to a significant increase in the haemolysis area for the iPPVO group compared to the control group (two-factor ANOVA test; * and ** for *p* < 0.05 and *p* < 0.005, respectively). A red star represents the significant increase in the haemolysis area for the control group over time, and the blue stars represent the significant increase in the haemolysis area for the iPPVO group over time.

Our objective with the study was to evaluate the effect of iPPVO injections on the serological response of horses to a semi-annual EI booster (SAB) vaccination. First of all, however, two groups of 10 horses with a similar serological response to the annual EI vaccination (AV) had to be assembled (Figure 1). The SRH assay was used to measure the level of EIV-specific antibodies. As the first group would receive the SAB vaccination alone 6 months after AV, it was designated as the “control group”, and as the second group would receive the SAB vaccination plus iPPVO injections, it was referred to as the “iPPVO group”. At the time of AV (Figure 2a,b), horses in both groups showed protective antibody levels against the two EIV strains (EIV A/equine/Paris/1/2018 and A/equine/Jouars/4/06 strains) used in the assay, with values > 85 mm^2^ (105.7 ± 18.8 mm^2^ and 141.9 ± 24.3 mm^2^, respectively, for the control group; 102.9 ± 21.8 mm^2^ and 129.7 ± 31.6 mm^2^, respectively, for the iPPVO group). No significant differences in SRH antibody titres were measured between groups when tested against the two EIV strains (*p*-value > 0.05). One month after the annual vaccination (AV + 1M), horses in both groups showed a significant increase in SRH antibody levels when compared with AV + 0M (154.4 ± 21.1 mm^2^ (*p*-value < 0.0001) and 171.3 ± 22.7 mm^2^ (*p*-value = 0.037), respectively, for the control group; 154.9 ± 24.3 mm^2^ (*p*-value < 0.0001) and 167.4 ± 27.7 mm^2^ (*p*-value = 0.0002), respectively, for the iPPVO group). SRH antibody titres were not significantly different between groups (*p*-value > 0.05). Three months after the annual vaccination (AV + 3M), the horses maintained a mean SRH value of 142.3 ± 18.7 mm^2^ and 151.7 ± 21.7 mm^2^ respectively, for the control group and 148.1 ± 17.7 mm^2^ and 151.2 ± 24.8 mm^2^ respectively, for the iPPVO group. No significant difference was measured when compared with AV + 1M or between groups (*p*-value > 0.05). At 6 months post annual vaccination (AV + 6M), all horses of the two groups maintained a protective level of SRH antibody when tested against the two EIV strains (A/equine/Paris/1/2018 EIV and A/equine/Jouars/4/06 strains). SRH antibody titres were 159.0 ± 28.8 mm^2^ and 161.1 ± 22.9 mm^2^, respectively, for the control group and 157.8 ± 23.6 mm^2^ and 154.2 ± 26.5 mm^2^, respectively, for the iPPVO group (Figure 2a,b). The differences between the two groups were not significant (*p*-value > 0.05). A two-way ANOVA using treatment (±IPPVO) and timing (from AV to AV + 6M) showed no significant difference between the two groups (*p*-value > 0.05 for SRH antibody titres tested against A/equine/Paris/1/2018 and A/equine/Jouars/4/06, respectively). Therefore, animals of the two groups showed a similar SRH response to the annual EI vaccination.

### 3.2. Antibody Response to EIV Vaccination with or without iPPVO as Immunostimulant

Horses received the SAB vaccination alone (control group) or together with iPPVO injections (iPPVO group). One month later (SAB + 1M), SRH antibody titres against A/equine/Paris/1/2018 were determined (Figure 2a) and did not significantly increase when compared with titres at the time of SAB in the control group (177.5 ± 18.9 mm^2^; *p*-value > 0.05). On the contrary, a significant increase was observed in the iPPVO group when comparing titres at the time of SAB vaccination and 1 month later (198.4 ± 16 mm; *p*-value < 0.0001). Additionally, horses in the iPPVO group showed SRH antibody titres significantly higher than those in the control group (*p*-value = 0.0375). Three months after the booster vaccination (SAB + 3M), mean SRH titres were 170 ± 22.6 mm^2^ and 185.7 ± 30.1 mm^2^ for the control and iPPVO groups, respectively. The difference between SAB + 1M and SAB + 3M were not significant, irrespective of the group (*p*-value > 0.05). Six months after the booster vaccination (SAB + 6M), the mean SRH value of the iPPVO group was 171.6 ± 20.4 mm^2^, while that of the control group was 155.3 ± 23.9 mm^2^, but the difference between the two groups was not significant (*p*-value > 0.05). A two-way ANOVA using treatment (±iPPVO) and timing (from SAB + 1M to SAB + 6M) showed a significant difference between the two groups (*p*-value = 0.004) for SRH antibody titres tested against A/equine/Paris/1/2018.

One month after the booster vaccination (SAB + 1M), SRH antibody titres against A/equine/Jouars/4/06 were also determined (Figure 2b). No significant increase was observed in the control group when comparing titres at the time of SAB and 1 month later (176.0 ± 10.8 mm^2^; *p*-value > 0.05). Titres significantly increased in the iPPVO group (196.3 ± 18.1 mm^2^; *p*-value < 0.0001). In addition, SRH antibody titres in the iPPVO group were also significantly higher than in the control group (*p*-value = 0.0439). Three months after the booster vaccination (SAB + 3M), mean SRH titres were 170.4 ± 19.15 mm^2^ and 192.5 ± 20.10 mm^2^ for the control and iPPVO groups, respectively. In addition, the difference between the two groups was significant (*p*-value = 0.0279). Between SAB + 1M and SAB + 3M, SRH titres did not significantly change, irrespective of the group (*p*-value > 0.05). Six months after the boost immunisation (SAB + 6M), the mean SRH value of the iPPVO group was 188.2 ± 18.2 mm^2^ while that of the control group was 162.1 ± 14.26 mm^2^, and the difference between the two groups was significant (*p*-value > 0.0099). A two-way ANOVA using treatment (±iPPVO) and timing (from SAB + 1M to SAB + 6M) showed a significant difference between the two groups (*p*-value < 0.0001) for SRH antibody titres tested against A/equine/Jouars/4/06. Therefore, iPPVO injections increased the serological response of horses to EI booster vaccination.

### 3.3. Individual Antibody Response to EIV Vaccination and iPPVO and Level of Protection

**Figure 3:**
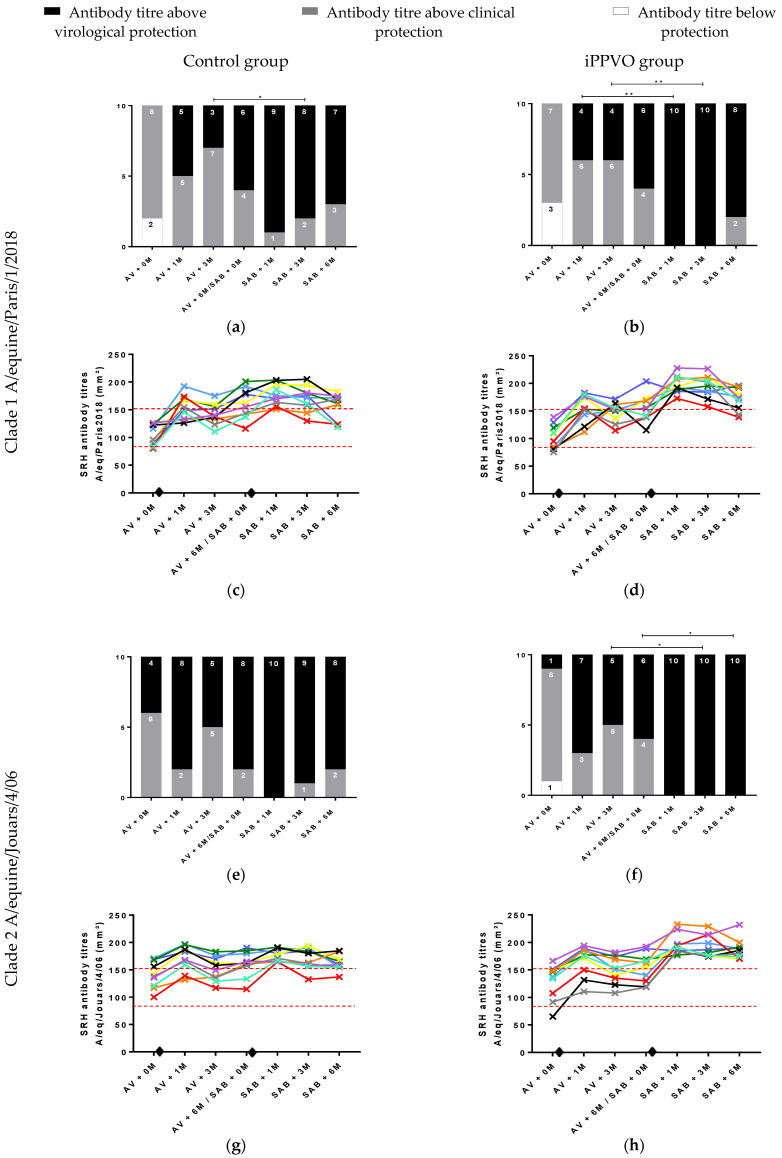
Evolution of H3N8 antibody response to simple radial haemolysis (SRH) measured before and after annual vaccination (AV) and semi-annual vaccination (SAB) with or without iPPVO. Values are for sera isolated from blood samples that were collected for 1 year from AV to 6 months after SAB. Values correspond to sera isolated from blood samples collected in the control and iPPVO groups before AV, 1 and 3 months after AV; before the booster, 1, 3 and 6 months after the semi-annual booster (SAB). Cumulative histograms represent the distribution of the number of horses according to the different levels of protection (white: unprotected; grey: clinical protection and black: virological protection) for the different sampling points for horses in the control group (**a**) and horses in the iPPVO group (**b**) for A/equine/Paris/1/2018 strain and in the control group (**e**) and horses in the iPPVO group (**f**) for A/equine/Jouars/4/06 EIV strain. Each horse is represented with a specific colour in the different graphs (c, d, g and h). The different colours correspond to the SRH values of the horses in the control group (*n* = 10) (**c**) and iPPVO group (*n* = 10) (**d**) for A/equine/Paris/1/2018 strain and the SRH values of the horses in the control group (*n* = 10) (**g**) and iPPVO group (*n* = 10) (**h**) for A/equine/Jouars/4/06 EIV strain. The diamond represents the timing of the AV injection and the 6 month SAB injections with or without iPPVO. * and ** indicate significant difference (χ^2^ tests; *p* < 0.05 and 0.005, respectively) in the frequency of horses reaching the protection threshold between the control group and the iPPVO group. Only similar time points post immunisation were compared.

Individual results for SRH titres determined with the A/equine/Paris/1/2018 strain are presented in Figure 3a–d. Before annual booster vaccination (AV), eight horses were above the clinical protection threshold (between 85 and 150 mm^2^), and two were below the protection threshold in the control group (<85 mm^2^) (Figure 3a,c) In the iPPVO group, seven horses were above the clinical protection threshold and three below the protection threshold (Figure 3b,d). After AV and before the semi-annual boost immunisation (SAB), no horse was below the clinical protection threshold in either group, and the number of horses above the virological protection threshold (>150 mm^2^) varied between three and six in the control group (Figure 3a,c) and between four and six in the iPPVO group (Figure 3b,d). After the semi-annual boost immunisation (SAB + 1M, 3M and 6M time point), no horse was below the clinical protection threshold in either group. In the control group (Figure 3a,c), the number of horses above the virological protection threshold decreased from nine to seven at the end of the study. In the iPPVO group (Figure 3b,d), the number of horses above the virological protection threshold decreased from ten to eight at the end of the study. When looking at the frequency of horses that reached the criteria for clinical or virological protection, no significant difference was observed between the two groups. However, for two time points (SAB + 1M and +3M), all horses in the iPPVO group had SRH antibody titres above the virological threshold, suggesting that all horses in this group were fully protected for at least 3 months. From an association point of view, there was a significant difference in the frequency of horses based on the protection threshold between the points AV + 3M and SAB + 3M (*p*-value = 0.025) for the control group (Figure 3a), which shows that significantly more horses passed the virological protection threshold 3 months after a semi-annual boost than at 3 months post annual booster. There was a significant difference in the frequency of horses based on the protection threshold between the points AV + 1M and SAB + 1M (*p*-value = 0.003), and AV + 3M and SAB + 3M (*p*-value = 0.003) for the iPPVO group (Figure 3b). These results show that significantly more horses passed the virological protection threshold at 1 and 3 months after a semi-annual boost than at 1 and 3 months post annual booster. 

Individual results concerning the A/equine/Jouars/4/06 strain are presented in Figure 3e–h. Before AV, six horses were above the clinical protection threshold, and four were above the virological protection threshold in the control group (Figure 3e,g). In the iPPVO group (Figure 3f,h) only one horse was below the clinical protection threshold, eight horses were above the clinical protection threshold, and only one was above the virological protection threshold. After AV and before the SAB immunisation, no horse was below the clinical protection threshold in either group, and the number of horses above the virological protection threshold varied between five and eight in the control group and between five and seven in the iPPVO group (Figure 3e,g). After the SAB immunisation, no horse was below the clinical protection threshold in either group. In the control group, the number of horses above the virological protection threshold decreased from ten to eight at the end of the study (Figure 3e,g). In the iPPVO group, all horses were above the virological protection threshold (Figure 3f,h). As above, when looking at the frequency of horses that reached the criteria for clinical or virological protection, no significant difference was observed between the two groups. However, for three time points (SAB + 1M, +3M and +6M), all horses in the iPPVO group were above the virological threshold. From an association point of view, there was no significant difference in the frequency of horses based on the protection threshold for the control group (Figure 3e). In contrast, there were significant differences in the iPPVO group between the AV + 3M and SAB + 3M (*p*-value = 0.010) and AV + 6M and SAB + 6M time points (*p*-value = 0.025) (Figure 3f). These results show that significantly more horses passed the virological protection threshold at 3 and 6 months after a semi-annual boost than at 3 and 6 months post annual booster.

## 4. Discussion

EIV vaccines are the most effective prophylactic treatments for controlling EI and preventing large-scale outbreaks. In order to maintain a good level of protection in the equine population, it is essential that a strong and sustained immune response is induced in all vaccinated individuals. Although current EI vaccines are still effective, there has been a delay in updating viral strains used in their composition, which presents an increasing risk of vaccine failure over time. In 2019, EIV infected a large number of horses in Europe, some unvaccinated and some vaccinated, but the clade 1 strain responsible for this outbreak [4] is believed to be more virulent, which may explain the scale of the crisis. Although this strain did not warrant an update of the vaccine following the WOAH recommendations [26], increasing protection by improving the vaccination protocol may provide an advantage over the virus and its dissemination. The current study showed that injections of iPPVO improved the humoral immune response of horses to booster vaccination against EI. This was assessed by quantification of antibody levels as determined by SRH assay. Several studies have demonstrated the correlation between the level of protection of horses and antibody levels measured by SRH. This method, using relevant EIV strains, allows for the accurate characterisation of the expected protection of the horse against EI. Results presented in this report suggest that iPPVO injection during EI vaccination could improve the level of protection of horses by increasing the level of SRH antibody induced by EI vaccination.

In this study, the choice of the equine participants was an important element of consideration. In the absence of information on the effect of iPPVO on influenza antibodies measured with the SRH assay and for greater robustness, a group of horses with homogeneous and similar SRH antibody responses to EI vaccination were chosen. A selection process based on the SRH antibody response to the standard annual EI vaccination allowed the constitution of small homogenous groups where the detection of the positive effects of iPPVO injections would be possible with a limited number of individuals.

Some studies have already shown the immunomodulation effect of iPPVO in vitro and in vivo on various infections in horses, cattle and pigs. To our knowledge, only one in vivo study using iPPVO as an immunostimulant has been conducted on horses in the context of EIV infection. The study by Lunn et al., in which horses were experimentally infected with EIV and treated or not with Baypamune^®^ (former trade name of iPPVO) showed that although the immunostimulant did not significantly reduce clinical signs, it significantly increased the antibody response to EIV infection of the treated horses when compared with control horses [27]. In the same study, the antibodies of 2 groups of horses were measured before, during and after 2 days of transport. It was shown that iPPVO improves the immune responses by SRH assay in immunosuppressed horses during transport. To date, no studies have been conducted on the use of iPPVO to improve EI vaccination. Results presented here showed for the first time that iPPVO co-injected with Equilis prequenza^®^ Te vaccine significantly increased the level of EIV-specific SRH antibodies when measured 1 and 3 months after vaccination. 

The Equilis prequenza^®^ Te vaccine used in this study did not contain an FC2 strain but induced similar SRH antibody levels when tested against FC1 and FC2 EIV strains. This observation had already been made in studies by Reemers et al. and Paillot et al., in which the Equilis prequenza^®^ Te vaccine conferred good clinical protection against recent field strains of EIV (FC2) [28,29].

In this study, a horse that did not show antibody levels sufficient for clinical protection prior to annual vaccination was considered a poor responder to vaccination. This study included three poor responders in the iPPVO group and two in the control group for FC1 and only one poor responder in the iPPVO group for FC2. Results show a potential ability of iPPVO to increase the immune response in a low responder horse, as defined above. If confirmed, this capacity would be interesting in order to boost the immune responses of horses, to limit the risk when horses are introduced into a horse population. The introduction of a sub-clinically infected horses could be the cause of epizootics such as the 2007 epizootic in Australia [6,30]. This observation further supports the use of iPPVO as an immunostimulant in the context of vaccination against EI. 

Two main limitations of this study have been identified: (i) the horses were all females from the same stud farm for reasons of availability and convenience in conducting the experimental study; (ii) the study included five low responders to FC1 stain and one low responder to FC2 strain. It will be interesting in a future study to replicate this work on a population of low responders in order to measure the potential of iPPVO when combined with an EI vaccine and its ability to mitigate the poor response to immunisation. Such characteristics would allow one to improve herd immunity and reduce the associated risks of an EI epizootic. It would also be interesting to test iPPVO in a population with high antibody levels to see if horses have individual or species-related limitations in their response to EI vaccination (i.e., is there a ceiling to the SRH antibody response induced by vaccination, is it frequently achieved). However, the SRH assay has its own limitations in measuring areas of haemolysis that may bias the quantification of high antibody titres. This study was only able to measure SRH responses 6 months post SAB. It would be interesting to follow the horses over a year to measure the iPPVO effect on the duration of immunity beyond 6 months. As this study was conducted with an inactivated EI vaccine, it would be interesting to define the effects of iPPVO when combined with EI vaccine using different technologies, for example, a canarypox vector vaccine, subunit vaccines or modified live attenuated viruses. Finally, it would be interesting to conduct a similar study with other equine vaccines such as the ones against equine herpesviruses (EHV1-4), pathogens that regularly cause epizootics in the equine populations.

Vaccines are composed of several components: one or more antigens specific to the infection against which protection is sought and adjuvants. One critical function of the adjuvants is to trigger the innate immune response that will prime the adaptive response against the antigen. At present, only a few compounds are authorised for use in human and veterinary medicine. Equine vaccines contain adjuvants that have been used for a long time, but some compounds are more recent and diversify the vaccine [31]. The EI vaccines available on the market mainly rely on the following adjuvants: aluminium salts, emulsions, carbomers, saponins and ISCOMs [2,7,31]. This study unravelled interesting properties of iPPVO, a substance approved in several countries as an immunostimulant but not as a vaccine adjuvant. It should be noticed that in this experimental protocol, iPPVO was not mixed and co-injected with the vaccine, but administered near the injection site of the vaccine. The near effect is probably due to the fact that both iPPVO and components of the vaccine converge on the same draining lymph node. In order to increase the panel of adjuvant substances available on the market and to maintain effective vaccines, it is warranted to study the effects of iPPVO when directly incorporated in a vaccine preparation, either alone or in addition to other adjuvants. In this study, Zylexis^®^ was used at half the dose recommended by the manufacturer for large animals. It is possible that the antibody response measured by SRH could be further enhanced at the dose that is actually recommended. However, co-injection with an adjuvanted vaccine is not documented, and it is not excluded that side effects may occur when combining the vaccine with a higher dose of Zylexis^®^. Future studies should address this point. Since iPPVO is derived from a virus (PPVO) that has undergone inactivation and is, therefore, not a chemically synthesised compound, this is an advantage over synthetic adjuvants, which are subject to controversy. This represents interesting perspectives for future studies. However, and most importantly, the current results show that iPPVO can be used in its commercialized form to boost the immunogenicity of existing EI vaccines without the need to reformulate either of them.

## Data Availability

Not applicable.

## Supplementary Materials

The following supporting information can be downloaded at: https://www.mdpi.com/article/10.3390/vaccines10122139/s1, Table S1: Rectal temperature (°C) and injection site effects before injection and 6 days after the first injection of iPPVO.
