# Supplementary material for: Immunostimulating Effect of Inactivated Parapoxvirus Ovis on the Serological Response to Equine Influenza Booster Vaccination
Source: Vaccines (Basel). 2022 Dec 14;10(12):2139. doi: 10.3390/vaccines10122139 (PMC9782193; doi:10.3390/vaccines10122139)
Supplement: Supplementary file 1 [file vaccines-10-02139-s001.zip › vaccines-2072846-supplementary.pdf]

Table S1. Rectal temperature (°C) and injection site effects before injection and 6 days after the first injection of iPPVO.

	**Horse n°**	**Injection -1D**	**Injection D**		**Injection +1D**		**Injection +2D**		**Injection +3D**		**Injection +4D**		**Injection +5D**		**Injection +6D**	
		Evening	morning**	Evening	morning	Evening	morning*	Evening	morning	Evening	morning*	Evening	morning	Evening	morning	Evening
	1	37.5	37.5	37.8	37.5	37.8	37.5	37.7	37.8	37.7	37.3	37.6	38	37.8	38	37.8
	2	38	37.6	38	37.8	38	37.7 swelling < 5 cm	37.6	37.9	38	37.7	37.9	38	38	37.7	38
	3	37.3	38	37.7	38	38	37.8	37.4	37.9	37.7	38.1	37.9	37.7	37.7	37.6	37.7
	4	36.7	37.3	38	37.1	37.9	37.4	37.2	37.4	37.4	37.3	37.4	37.4	37.6	37.3	37.6
**Control Group**	5	37.1	37.4	37.7	37.6	37.6	37.4	37.2	37.3	37.2	37.3	37.5	37.2	37.4	37.2	37.6
	6	37.1	37.3	37.7	37.4	37.7	37.4	37.7	37.7	37.8	37.7	37.7	37.7	37.7	37.4	37.5
	7	36.6	37.7	37.9	37.7	38	37.8	37.7	37.2	37.5	37.4	37.6	37.3	37.6	37.7	37.7
	8	37.1	37.4	37.9	37.8	37.7	37.7 swelling < 5 cm	37.4	37.4	37.7	37	37.6	37.2	37.3	37.4	37.7
	9	37	37.3	38.1	37.6	37.9	37.5	37.5	37.6	37.6	37.3	36.9	37.4	37.7	37.4	37.7
	10	37.1	37.4	38	37.3	37.4	37.9	37.7	37.5	37.5	37.7	37.1	37.1	37.1	37.4	37.7
	11	36.9	37.3	38	36.9	37.4	37.7	37.6	37.7	37.7	37.5	37.4	37.7	37.4	37.8	37.7
	12	36.9	37.4	37.6 swelling 7 cm	37.9 swelling 10 cm	38	37.3	37.7	37.4	37.8	37	36.6	37.7	37.1	37.2	36.4
	13	37.3	37.9	38.4	37.9	38.5	37.9	38	38.2	37.8	37.5	37.8	37.8	38.2	37.8	38
	14	36.7	36.7	37.6	37.3	37.7	37.6	36.9	37	37.5	37.2	37.8	37.2	37.5	37.5	37.5
**iPPVO group**	15	38	37.8	38.1	37.3	38.1	37.9	37.8	37.7	37.8	37.4	37.7	37.4	37.9	37.9	38.2
	16	37.3	37.3	37.9	37.4	37.9	37.4	37.4	37.5	37.5	37.4	37.4	37.7	37.8	37.5	37.8
	17	36.7	37.7	37.8	37.8	37.9	37.3 swelling < 5 cm	37	37.8	37.6	37.4	37.2	nc	37.3	37.6	37.8
	18	37.3	37.6	37.6	37.7	38.1	37.4	37.6	37.5	37.6	37.7	37.6	37.8	37.3	37.1	37.7
	19	37.6	38	38.4	37.7	38.1	38	38	38	38.1	38.1	38	38	38.2	37.9	38
	20	37.3	37.4	38	37.7	37.9	37.3	37.5	37.8	37.7	37.1	37.9	37.7	37.7	37.7	38

*iPPVO injection
**co-injection of iPPVO and EI vaccination
